# Undifferentiated embryonal sarcoma masquerading as liver abscess: A case report with typical imaging features

**DOI:** 10.4102/sajr.v25i1.2179

**Published:** 2021-08-31

**Authors:** Ranjan K. Patel, Swasti Pathak

**Affiliations:** 1 Department of Radiodiagnosis, Maulana Azad Medical College, New Delhi, India; 2 Department of Interventional Radiology, Institute of Liver and Biliary Sciences, New Delhi, India

**Keywords:** undifferentiated embryonal sarcoma, paradoxical appearance, fever, liver abscess, serpiginous vessels, haemorrhage

## Abstract

Undifferentiated embryonal sarcoma (UES) is an uncommon paediatric hepatic tumour that clinically simulates a liver abscess when present with fever. This report describes a case of UES in a 12-year-old boy, who presented with abdominal pain, swelling and fever, all simulating a liver abscess. The possibility of UES was considered at imaging, based on the solid appearance on ultrasound and cystic appearance with serpiginous peripheral vessels on computed tomography/magnetic resonance imaging. The diagnosis was confirmed at histopathology.

## Introduction

Undifferentiated embryonal sarcoma (UES) of the liver is a rare malignant hepatic tumour of mesenchymal origin. It is primarily seen in children between 6 and 10 years of age.^1^ Clinical manifestations are often non-specific, including abdominal pain and swelling, hepatomegaly, decreased appetite and weight loss. Additionally, some patients present with intermittent and low-grade fever, making it difficult to distinguish from a liver abscess or a complicated hydatid cyst.^2^ However, an accurate diagnosis is essential to initiate appropriate management. Imaging features can be variable; however, a peculiar paradoxical appearance on imaging, that is, solid on ultrasonography (USG) and cystic on CT or MRI, can help in the early diagnosis of this entity.^3,4^ Presented here is a case of UES of the liver in a 12-year-old boy who presented with abdominal pain, right-sided upper abdominal bulge and low-grade fever, masquerading as a liver abscess.

## Case report

A 12-year-old boy was admitted with complaints of mild abdominal pain and fever for one month. The intermittent and low-grade fever was relieved with analgesics. The mild and dull aching abdominal pain was confined to the right hypochondrium without any radiation. He also complained of a right upper abdominal bulge. There was a history of decreased appetite and weight loss of about 2–3 kg. The patient was haemodynamically stable. On abdominal examination, a firm and mildly tender mass was palpable 10 cm below the right costal margin, and the mass was not separately palpable from the liver. No prior history of hydatid disease, malignancy or any other comorbidity was found.

**FIGURE 1:**
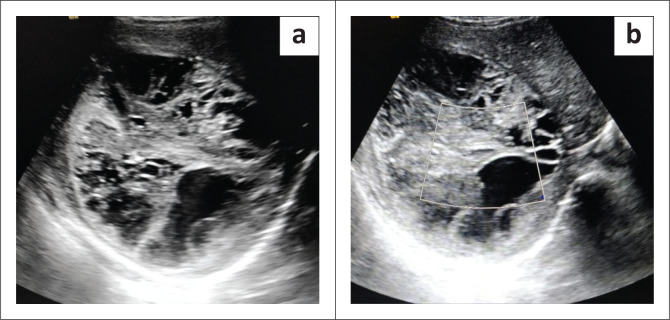
(a) A well-defined heterogeneous lesion in the right lobe of liver with solid echogenic areas and anechoic cystic areas; (b) No obvious internal vascularity on Doppler imaging.

At USG, a large well-defined mass was found in the right lobe of the liver. It was solid and iso to hyperechoic in appearance with a few cystic areas (Figure 1a). No apparent internal vascularity was demonstrated on the colour Doppler study (Figure 1b). Based on the fever and USG findings, the possibility of an organised liver abscess or complicated hydatid cyst was considered. Empiric IV antibiotics and albendazole therapy were initiated. Liver function tests (liver enzymes, serum bilirubin and albumin levels) and complete haemogram were within the normal limits except for a low haemoglobin of 9 gm/dL. Total leucocyte counts and eosinophil counts were normal. Both hydatid (immunoglobulin G [IgG] titre-0.7; normal < 0.9) serology and amoebic serology (IgG titre-0.51; normal < 0.9) were non-reactive. Tumour markers were also within the normal limits. Serum alfa-fetoprotein (AFP), beta-human chorionic gonadotropin (beta-hCG) and carbohydrate antigen (CA-19-9) were 3.2 ng/mL (0–8.5), 0.8 IU/L (< 1.4) and 14 IU/mL (< 35), respectively.

**FIGURE 2:**
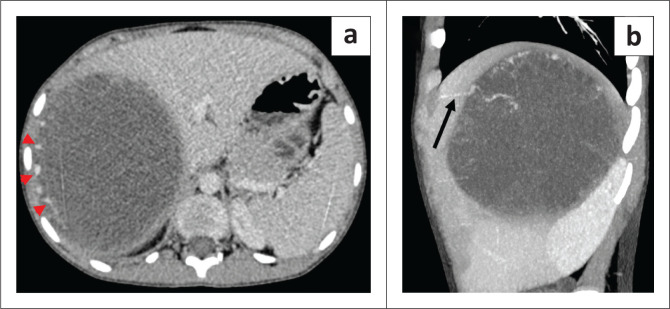
(a) Axial contrast-enhanced CT images demonstrating the cystic appearance of the mass with eccentric internal vascularity (red arrow heads); (b) Sagittal maximum intensity projection image showing a tortuous artery supplying the periphery of the lesion as denoted by the black arrow.

**FIGURE 3:**
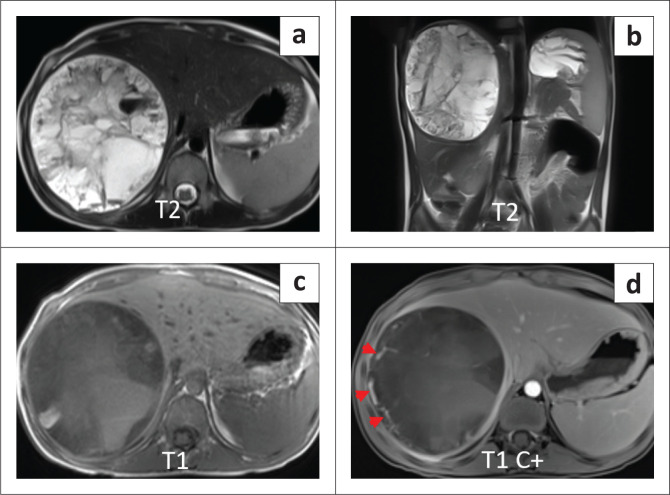
(a) Axial and (b) coronal T2W images showing a hyperintense cystic appearing lesion with heterogeneous internal components and fluid-fluid levels; (c) Axial T1W image revealing hyperintense, internal haemorrhagic contents; (d) Post-contrast T1W image indicating only peripheral vascularity (red arrow heads) with no obvious internal solid enhancing soft tissue.

**FIGURE 4:**
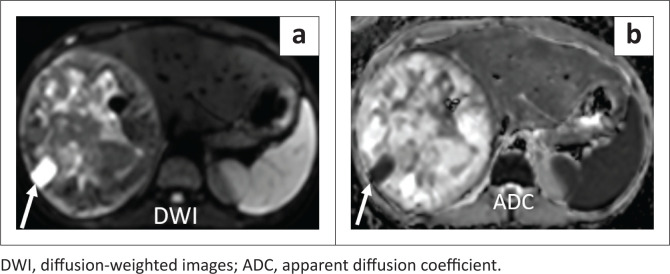
(a) Axial diffusion-weighted images image at *b* = 800 sec/mm^2^ shows heterogeneous content with hyperintensity areas that reveal low signal on the corresponding apparent diffusion coefficient map (b), indicating diffusion restriction. This was related to the internal haemorrhage and necrotic tissue. White arrows mark a focal haemorrhagic focus (*marked T1 hyperintensity as seen in Figure 3C*), showing significant restricted diffusion.

Following failure to respond to antibiotic therapy, contrast-enhanced CT (CECT) and CE-MRI were requested for further evaluation of the lesion. Contrast-enhanced CT demonstrated a cystic appearing mass (attenuation: 20–25 HU) with peripheral internal vascularity (Figure 2a and b). MRI revealed T2 hyperintensity with heterogeneous internal content and fluid-fluid levels (Figure 3a and b). Areas of variable T1 hyperintensity were also seen, possibly due to haemorrhagic components of varying ages (Figure 3c). No enhancing solid tissue was seen within the lesion on post-contrast (gadopentetate dimeglumine) T1 weighted (T1W) images except for a few peripheral enhancing vessels as seen on CECT (Figure 3d). Diffusion-weighted images (DWI) revealed variable signal with foci of restricted diffusion (Figure 4a and b), corresponding to the haemorrhagic and necrotic areas.

A core needle biopsy was performed under USG guidance, using an 18G semi-automatic gun; however, the pathology was inconclusive because of the predominant necrotic material obtained during the biopsy procedure. Microbiological analysis of the biopsy sample was negative for bacteria, fungi, Entamoeba and parasites. Due to the non-resolving condition and inconclusive diagnosis, the patient finally underwent a right hepatectomy with removal of the lesion approximately 16 days after hospitalisation. The resected specimen revealed a large soft and friable tumour. The cut surface of the specimen revealed components of haemorrhage, necrosis and gelatinous material. There were no intra-operative or postoperative complications, and the patient was discharged on the 11th postoperative day in a stable condition.

Histopathological analysis of the resected specimen indicated that the tumour was composed of sheets of spindle cells with cellular pleomorphism, indistinct cytoplasm and hyperchromatic nuclei. Immunohistochemistry showed CD10 membrane positivity, vimentin reactivity in spindle cells and negativity for Myo D1. These findings were suggestive of UES.

Postoperatively, the patient received six cycles of chemotherapy (vincristine + cyclophosphamide + epirubicin). He was asymptomatic with no recurrence on the last follow-up at 11 months after surgery.

## Discussion

Undifferentiated embryonal sarcoma is an uncommon malignancy of undifferentiated mesenchymal cells. It accounts for only 0.1% of surgically resected primary liver masses.^1^ Undifferentiated embryonal sarcoma is most commonly seen between 6 and 10 years of age; however, it has been reported in adult patients as well.^5^ Clinical manifestations are usually non-specific. Children present with abdominal pain, loss of appetite and weight and a mildly tender abdominal lump. Some patients may have fever and elevated leukocyte counts which cause difficulty in differentiating it from other hepatic pathologies, such as a liver abscess or complicated hepatic hydatid cyst.^2,3^ In a recent case series of 10 patients, fever was one of the manifestations in eight of 10 patients.^6^ A few patients present to the emergency department with acute abdominal pain because of tumour rupture and bleeding.^2^

Unlike other common paediatric hepatic tumours such as hepatoblastoma and hepatocellular carcinoma (HCC), AFP levels are usually not elevated in UES, which is an additional clue to its diagnosis. Alternatively, LDH levels can be elevated in about 70% – 75% of cases; however, the cause is still unclear.^2,3,6^

The imaging appearance of UES may be inconsistent, varying from entirely cystic to entirely solid.^7^ Characteristically, imaging shows a paradoxical appearance. On USG, UES shows solid-dominant mixed echogenic areas with variable anechoic cystic areas; however, it often appears as a cystic lesion with low attenuation on CT. This discrepancy in appearance is attributed to myxoid tissue, which usually appears hyperechoic on USG.^3,4^ A similar appearance was also seen in the patient presented. At times, lesions may be predominantly anechoic with multiple septa, simulating benign tumours such as mesenchymal hamartoma and biliary cystadenoma.^8^ Nodular solid components and septations are also not uncommon, which show delayed enhancement.^3^ This case showed serpiginous vessels along the periphery of the lesion which has also been described in a previous study by Gabor et al. Serpiginous vessels were found in 9 of 15 patients in their study.^3^ Undifferentiated embryonal sarcoma often shows areas of haemorrhage, and MR signal varies depending on ages of the bleeds. Hyperintense signal on T1W images with fluid-fluid levels can occur because of internal haemorrhage and necrosis. Haemorrhagic ascites or perihepatic fluid may be seen in cases of tumour rupture.^3,8^ Neither was found in our patient.

Mesenchymal hamartoma is the most common imaging differential as both share common imaging features. Although age may not be a reliable element, age < 2 years usually favours mesenchymal hamartoma and age > 5 years, usually favours UES. Additionally, intralesional haemorrhage and serpiginous vessels favour UES.^8^ Rhabdomyosarcoma is an alternative differential; however, it arises from the biliary tree and causes biliary obstruction, unlike UES.^8^

Clinically and radiologically, UES simulates a liver abscess, particularly when fever and leukocytosis are present.^6^ Another lesion that closely mimics UES on imaging is a hydatid cyst, especially in endemic regions. A complicated hydatid cyst can present with fever.^9^ Amoebic serology, hydatid serology and fluid analysis may provide clues to the specific diagnosis.

Hepatoblastoma and HCC rarely have a cystic appearance; however, AFP levels are often elevated in these tumours, unlike UES, where the AFP level is often normal.^8^ A hepatic tumour in a child < 5 years old that shows lower enhancement than the adjacent hepatic parenchyma and elevated serum AFP levels is more likely to be hepatoblastoma. Hepatocellular carcinoma usually arises on a background of diffuse parenchymal liver diseases, such as glycogen storage disorders, tyrosinemia, Wilson’s disease, etc.^10^ Hepatocellular carcinoma appears as an arterially hyperenhancing mass with washout during the portal venous or delayed phases of imaging. It is commonly seen in children > 5-year-old.^8,10^ Various salient features of the differential diagnoses of UES are summarised in Table 1.^2,3,4,6,8,9,11,12^

**TABLE 1:** Differential diagnoses of Undifferentiated Embryonal Sarcoma of liver.

Differential diagnosis	Clinical and laboratory features	Imaging features
Undifferentiated embryonal sarcoma (UES)	Age > 5 years; fever; tender hepatomegaly; palpable lump;	Paradoxical appearance:
	leukocytosis; normal AFP	Solid on USG; cystic on CT/MRI; intralesional haemorrhage and serpiginous vessels
Mesenchymal hamartoma	Age < 2 years; abdominal lump	Multiseptated cystic lesions interspersed with solid components; septae and solid tissue showing enhancement
Liver abscess	Fever; pain; tender hepatomegaly	Peripheral enhancement; no solid enhancing components
	Leukocytosis; positive amoebic serology or bacterial culture	
Hydatid cyst	Palpable lump; abdominal heaviness; fever in infected cyst;	Cystic lesion with or without daughter cysts; floating internal membrane; T2 hypointense peripheral rim
	positive hydatid serology; eosinophilia	
Hepatoblastoma	Age < 5 years; palpable lump; abdominal pain; jaundice;	Well-defined mass; heterogeneously hypoenhancing to normal liver; speckled/amorphous Ca^+2^ in 50% cases
	Elevated AFP (90% cases)	
Hepatocellular carcinoma	Age:10–14 years; pre-existing liver diseases in 30% – 50% cases;	Arterially hyperenhancing with portal venous or delayed phase washout; presence of tumour capsule
	Elevated AFP (70% cases)	
Infantile hepatic haemangioma	Age < 1 year; abdominal lump; high output cardiac failure; thrombocytopenia; normal AFP	Peripheral, nodular enhancement on arterial phase with gradual centripetal filling on delayed phase; fine and granular calcification; enlargement of hepatic artery; tapering of abdominal aorta below celiac axis
Biliary rhabdomyosarcoma	Age < 3 years; abdominal lump; obstructive jaundice	Mass along the biliary tree; heterogeneous and hypoenhancing; dilated intrahepatic biliary radicles

*Source*: Please see the full reference list of the article Wei ZG, Tang LF, Chen ZM, Tang HF, Li MJ. Childhood undifferentiated embryonal liver sarcoma: Clinical features and immunohistochemistry analysis. J Pediatr Surg. 2008;43(10):1912–1919. https://doi.org/10.1016/j.jpedsurg.2008.06.016, for more information

AFP, alfa-fetoprotein; CT, computed tomography; MRI, magnetic resonance imaging; USG, ultrasonography.

Collection of inadequate specimens or sampling error usually leads to an incorrect histological diagnosis which often occurs in this condition because of the predominance of tumour necrosis and haemorrhage.^13^ Image guidance helps procure a biopsy sample from the viable part of the tumour with a higher diagnostic yield and is also associated with a lower complication rate. An 18G biopsy needle suffices in most instances.^3^ However, because of the predominantly cystic nature of UES, percutaneous biopsy may not provide representative tissue and an open biopsy may be required for diagnostic confirmation.^13^ A definitive diagnosis of UES is made only on histopathology, supported by immunohistochemistry.^7^

In the past, the postoperative recurrence rate was high. However, advancement in current treatments has substantially improved the clinical outcome with a lower rate of recurrence.^14,15^ Although there is no universal consensus regarding the management guidelines, complete resection of the tumour and combined adjuvant chemotherapy is the standard treatment strategy.^13,15^ Liver transplantation may be an option for patients with unresectable tumours. Imaging plays a vital role in post-treatment follow-up. It helps in the assessment of treatment response and to detect tumour recurrence early.^10,13^

## Conclusion

The peculiar paradoxical appearance of a paediatric liver lesion, that is, solid on USG and cystic on CT/MRI, should raise the possibility of UES, especially in the presence of normal AFP levels, serpiginous vessels and intralesional haemorrhage.
